# The use of community-engagement approaches to identify strategies for increasing workplace well-being among LGBTQ+ adults

**DOI:** 10.1016/j.heliyon.2024.e39541

**Published:** 2024-10-18

**Authors:** Nanchatsan Sakunpong, Ramida Mahantamak, Pilaiporn Sukcharoen, Alicia K. Matthews

**Affiliations:** a Behavioral Science Research Institute of Srinakharinwirot University, Bangkok, Thailand; b Mahidol University International Demonstration School, Nakhon Pathom, Thailand; c Faculty of Nursing, Suratthani Rajabhat University, Suratthani, Thailand; d School of Nursing, Columbia University, New York, USA

**Keywords:** Well-being, LGBTQ+, Workplace, Qualitative methods, Community-engagement

## Abstract

**Background:**

LGBTQ + individuals often face social stigma and workplace discrimination, negatively impacting their well-being. However, limited research exists on the factors and strategies that enhance their positive workplace experiences. This study was a sequential qualitative mixed-method design conducted in three phases. The study had the following objectives: identifying factors associated with workplace well-being, generating strategies for improving workplace well-being, and obtaining feedback on the identified strategies' acceptability.

**Methods:**

In Phase 1, in-depth interviews were conducted with 10 LGBTQ + who described factors that improved their satisfaction at work. In Phase 2, two focus group discussions were held with 13 participants, including LGBTQ + adults, scholars in positive psychology, human resource development, and civil rights advocates, to develop strategies for improving workplace well-being based on Phase 1 data. In Phase 3, a survey was sent to a subset of the focus group participants (N = 7) to ascertain the acceptability of the developed strategies. Data analysis was performed using deductive and inductive approaches.

**Results:**

The study's first phase identified the factors contributing to workplace well-being. These factors were categorized into intrapersonal, interpersonal, and social-ecological. In the second phase and third phase, 11 strategies were developed to improve workplace well-being. These strategies were divided into two sections. The first section includes strategies organizations and agencies can implement to promote employee well-being (such as emphasizing diversity and equity as core values). The second section outlines strategies for government and civil society sectors (such as establishing laws addressing LGBTQ + equal rights). The Index of Congruence (IOC) assessed the relationship between strategies and workplace well-being improvement. Results showed IOC values from 0.8 to 1 for 11 strategies, indicating high content validity.

**Conclusions:**

Strategies to enhance LGBTQ + workplace well-being were identified through community engagement, but further research is needed for implementation and evaluation.

## 1 Introduction

Research has extensively highlighted the profound challenges encountered by lesbian, gay, bisexual, transgender, questioning, and other identities (LGBTQ+) throughout their lives [1]. Challenges experienced by LGBTQ + individuals encompass a range of adversities, including parental rejection, peer bullying in both community and educational settings, barriers to accessing adequate healthcare, and instances of exclusion and discrimination within the workplace [2,3]. These challenges stem from diverse sources, such as entrenched social, religious, and cultural norms, that lead to the marginalization of LGBTQ + individuals [4]. Moreover, the absence of legal protections contributes to sustaining biased and discriminatory attitudes directed toward members of these communities [5].

LGBTQ + individuals in Asia face significant workplace discrimination and stigma, impacting their well-being and professional growth [6]. Studies indicate frequent bias and exclusion from colleagues and supervisors. For instance, a report by the International Labour Organization (ILO) highlights that many LGBTQ + employees in Asia experience verbal harassment, unequal treatment, and barriers to career advancement due to their gender and sexual minority status [7]. Research in countries like China and India has shown that such discrimination leads to higher levels of stress, job dissatisfaction, and mental health issues among LGBTQ + employees [6]. Despite legal advancements in some regions such as the existence of equal marriage laws in Taiwan, societal attitudes remain conservative, perpetuating a culture of silence and fear. This environment hampers professional opportunities and affects the overall quality of life. Addressing these issues requires more efforts from policymakers and employers to foster inclusive workplace cultures and implement anti-discrimination policies [8].

In Thailand, a growing body of research underscores the pervasive discrimination faced by a significant portion of LGBTQ + individuals across various spheres, such as education, professional training, employment, and career advancement [9]. Additionally, these individuals often encounter obstacles in accessing vital employee welfare benefits, including spousal health insurance, and regularly confront job refusals [10]. Social discrimination contributes to a pattern where LGBTQ + individuals feel compelled to conceal their sexual or gender identities to secure job opportunities and maintain job security [11]. These observations emphasize the detrimental impact of the absence of explicit policies within Thai society aimed at addressing the treatment of LGBTQ + individuals, particularly in fostering workplace inclusivity and well-being for this demographic [9,12].

To date, most studies on LGBTQ + individuals in the workplace have primarily focused on investigating workplace discrimination and harassment. In the case of studies conducted in other countries, Ozeren [13] conducted a systematic review of 52 studies examining workplace discrimination among the LGBTQ + population. The review revealed that LGBTQ + individuals faced inequity, including salary inequities, the lack of spousal/partner benefits, and social exclusion by coworkers. Furthermore, Lloren and Parini [14] investigated policies to reduce sexual discrimination against LGBTQ + employees. The policies reviewed included the inclusion of a written non-discrimination clause about sexual orientation and gender identity within the company's charter; providing health insurance coverage for the domestic partners of employees of the same sex; implementation of a warning system and disciplinary actions to deter homophobia; establishment of mentoring and training initiatives focused on promoting LGBT equality and inclusion; and acknowledgment and endorsement of an LGBT network/group and an LGBT contact person within the organization. The authors also found that despite workplace policies meant to deter discrimination, many LGBTQ + employees continued to experience unfair treatment due to the lack of appropriate workplace policies being addressed, directly impacting their psychological well-being.

In a collaborative effort between the World Bank and Thammasat University, a study involving 2302 LGBTQ + individuals was conducted in Thailand [10]. The research revealed striking statistics: 77 % of transgender individuals, 62.5 % of lesbians, and 49 % of gay individuals reported being denied employment opportunities due to their LGBTQ + status. Additionally, 40 % of transgender individuals reported experiencing sexual harassment or being exposed to sexual jokes in the workplace. Moreover, 22.7 % of gay individuals reported being passed over for promotions because of their LGBTQ + identity, while 24 % of lesbians and gays were unable to disclose their sexual orientation in their workplace. Finally, 23.7 % of transgender individuals were compelled to use restrooms corresponding to their assigned sex at birth. These findings underscore significant barriers to fair job opportunities, emotional security, and well-being for LGBTQ + individuals in the workplace, suggesting a need for further research into factors that might enhance these outcomes.

A robust literature describes factors that improve employee well-being in the workplace [15,16]. Bartels, Peterson, and Reina [15] defined workplace well-being as employees' perception of their ability to enhance their functioning at work. These authors examined indicators of workplace well-being with a diverse sample group to develop a scale measuring workplace well-being. The research findings indicate that the exploratory factor analysis of the workplace well-being scale comprises the interpersonal and intrapersonal components when tested with a sample group of 120 university postgraduate students. This result substantiates the proposition that workplace well-being encompasses both the intrapersonal and interpersonal dimensions, as posited in the research hypothesis [15]. However, these studies primarily explored well-being in the context of individuals who are not specifically LGBTQ+. The primary aim of our study was to identify factors associated with workplace well-being, generate strategies for improving workplace well-being, and obtain feedback on the identified strategies' acceptability. Moreover, this study's objectives aligned with the United Nations Sustainable Development Goal (SDG) centered on fostering equality among LGBTQ + individuals. By addressing this knowledge gap, the study sought to bolster LGBTQ + well-being, contributing to the broader goal of promoting inclusivity and equality in work environments.

## 2 Research methodology

### 2.1 Study design

**Fig. 1:**
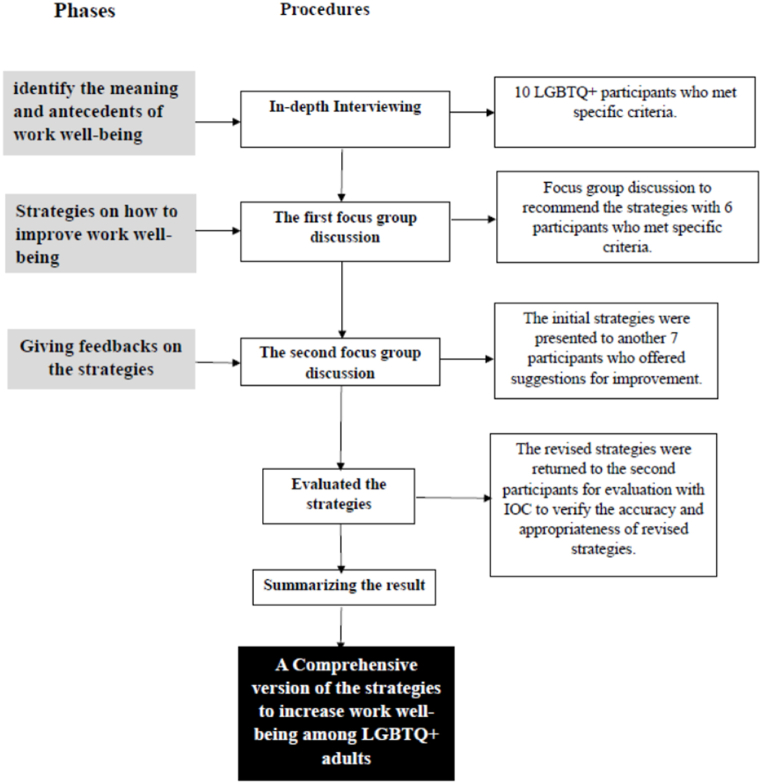
The sequential qualitative mixed method designs.

This study utilized a sequential qualitative mixed-method design [17], as illustrated in Fig. 1. Qualitative methods explore complex social phenomena and capture the subjective experiences, perspectives, and meanings that individuals attribute to their lived realities. The sequential qualitative mixed-method design involves interconnected stages of qualitative study, such that research findings from the initial stage can be utilized in the subsequent stage, and results from the second stage can be further applied in the third stage of the research. The Institutional Review Board of Srinakharinwirot University (SWUEC-162/2563E) reviewed and approved the study protocols and procedures on May 25, 2020. The research process steps are explained in detail in the section below.

### 2.2 Conceptual framework

**Fig. 2:**
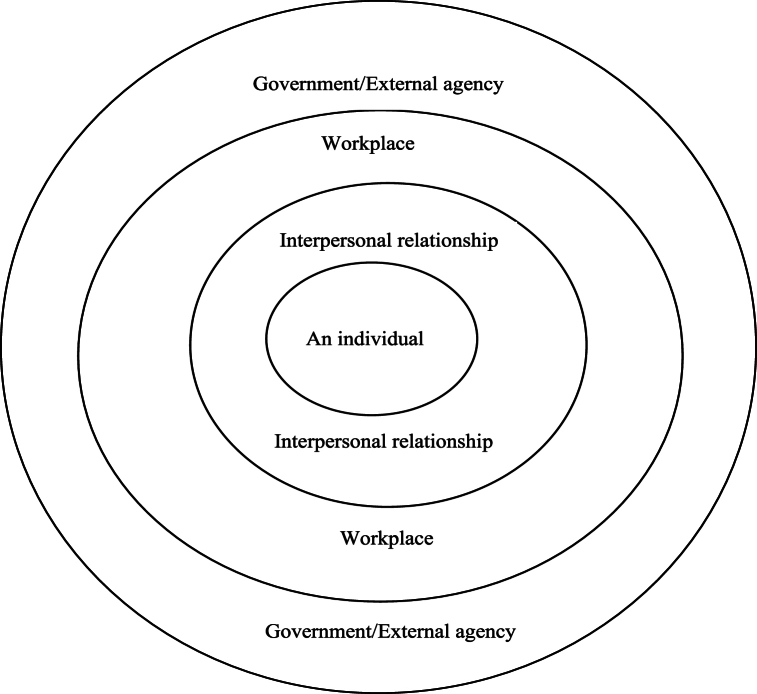
Conceptual framework of work well-being in the context of Thai LGBTQ + people.

The study's interview guides were developed using two primary frameworks. Initially, Bartel's concept of workplace well-being served as the cornerstone for crafting the in-depth interview questions [15]. This concept highlights the significance of both interpersonal and intrapersonal factors in shaping well-being within a workplace context. Moreover, the social-ecological framework [18] was used to refine the interview questions further. This framework challenges conventional psychological theories by emphasizing contextual factors in understanding individual behaviors. It underscores the need to consider a holistic exploration of multiple levels, encompassing interpersonal interactions, organizational dynamics, and societal dimensions beyond the organization's boundaries [18]. A visual representation illustrating the integration of these two theoretical frameworks is depicted in Fig. 2.

### 2.3 Research team and stance

The research team consisted of gender minority women, cis-gender women, and non-binary individuals with doctoral degrees and expertise in qualitative research. All work in educational settings and have experience in qualitative research for at least 3 years. Before the fieldwork, team meetings, and training sessions were conducted to ensure a shared understanding of the research procedures among all team members. Regular team meetings were held throughout the research process to validate the data analysis derived from interviews with each key informant, thereby enhancing the accuracy of the interpretation of the informant experiences.

The researcher's stance in this study aligns with the constructivist paradigm, which postulates that knowledge is multifaceted and emerges from integrating interpretations made by researchers and key informants during fieldwork [19]. This paradigm recognizes that knowledge is not an objective entity waiting to be discovered but rather a socially constructed understanding shaped by the perspectives and experiences of individuals involved in the research. By adopting a constructivist perspective, the researcher acknowledges that multiple realities and interpretations exist and are contextually situated and influenced by the social, cultural, and historical contexts in which they are embedded [20].

Following the constructivist paradigm, the researcher acknowledges the importance of employing various flexible and adaptable approaches to access knowledge. This study utilizes qualitative research methods, including in-depth interviews, focus group discussion, observation, and field notes, to gain a deep and nuanced understanding of the phenomenon under investigation [21,22].

### 2.4 Phase 1

The purpose of phase 1 was to investigate the meaning and antecedents of workplace well-being, which has details of the research methodology as follows.

#### 2.4.1 Participants

This study phase used in-depth interviews with 10 LGBTQ + employees working in various public and private agencies in Thailand. Announcements posted on Facebook were used to recruit participants for one month. Interested individuals are encouraged to contact the research team through various channels, such as the Line application, Facebook inbox, or email. Following study enrollment, participants completed a well-being-at-work screening checklist [15]. Individuals who obtained an overall mean score at a high level or above, indicating a high level of work well-being, were chosen as purposive participants in the study. Additional eligibility criteria included self-identity as LGBTQ+, employment, and age 22 to 60. These 10 participants provided qualitative data on the antecedents of work well-being within the context of LGBTQ + individuals.

#### 2.4.2 Research instrument the research instrument used in Phase 1 consists of 2 tools, as follows

1) **Well-being at work screening checklist.** The checklist was translated from the validated scale developed by Bartels, Peterson, and Reina [15]. The objective was to identify participants reporting high levels of workplace well-being for inclusion in Phase 1 of the study. The 8-item checklist utilized a 4-point Likert scale, ranging from the highest to the lowest, with corresponding scores of 4, 3, 2, and 1, respectively. A mean score of 3.00 or above indicated high levels of work well-being. This screening test was employed to identify information-rich cases subsequently invited to participate in the study. This checklist was used for all three phases of the study.
2) **In-depth interview questions.** A semi-structured interview guide was developed based on relevant concepts of workplace well-being [15,16,23]. The interview guide aimed to elicit participant insights regarding experiences within the workplace. Example questions included: “How do you define work well-being from your perspective?” “Which personal characteristics of LGBTQ + individuals do you believe contribute to work well-being?” and “How does society or the community impact the well-being of LGBTQ + individuals in the workplace?” Additional probes were used when appropriate to delve deeper into the participants' experiences. The questions aimed to facilitate the emergence of information through dyadic discussions, and the obtained data was subsequently subjected to qualitative analysis.

#### 2.4.3 Research procedure

Phase 1 recruitment consisted of an advertising strategy utilizing an information graphic (infographic) shared on the principal investigator's personal Facebook account. Individuals interested in participating could apply to the project through various channels, including LINE, email, and Facebook chat. Subsequently, the principal investigator conducted the interviews via Google Meet, and an audio recording was carried out to ensure accurate data capture. Each interview began with the researcher building rapport with the participants and then moving on to the prepared questions. The interviews lasted 60–90 min, allowing in-depth exploration of the informants' experiences. The collected data were then analyzed to uncover the definitions and antecedents of work well-being among LGBTQ + individuals. The research team contacted the participants for the second and third phases of the study by purposeful selection based on the study inclusion criteria. Group discussions were conducted using Google Meet to analyze the data. The data from the first phase was presented to the first group of six participants to generate strategies to improve work well-being in the LGBTQ + context.

#### 2.4.4 Data analysis

In the qualitative data analysis, the interviews were transcribed verbatim by the research team. The process combined deductive and inductive approaches. A deductive approach was initially used to generate main themes based on pre-existing theoretical frameworks. Subsequently, an inductive approach was employed to generate new codes and categories that supported each identified theme, allowing for new insights. This involved open coding to identify significant phrases from the transcribed data to address the research objectives, categorizing these codes into complementary or new sub-themes, and refining the initial themes to reflect the actual data. A constant comparison method was used to ensure consistency and thoroughness in capturing theoretical perspectives and novel insights from the data [24]. This qualitative data analysis was conducted manually without the use of any software.

A rigorous validation was undertaken to ensure the findings' validity and reliability. Three researchers with expertise in qualitative research independently read the interview transcripts and conducted their analyses of the same dataset. Following individual analyses, the researchers compared and contrasted their findings. In cases where inconsistencies or discrepancies arose, discussions were held among the researcher and the two analysts to reach a consensus. Only the analyses that obtained agreement among all parties were included in this study, enhancing the rigor of the findings. Additionally, the data analysis results from the research participants were disseminated to all participants for verification of the accuracy of the analysis, confirming that the findings accurately reflect the experiences of the research participants [25].

### 2.5 Phase 2

The purpose of phase 2 is to identify how to improve workplace well-being, which has details of the research methodology as follows.

#### 2.5.1 Participants

Six participants were purposively recruited for the Phase 2 focus group discussion. The participants included LGBTQ + employees with above-average workplace well-being scores (N = 2), a scholar focused on human resources development (N = 1), scholars specializing in positive psychology (N = 2), and a human rights and social welfare activist with expertise in gender and social welfare issues (N = 1). These participants provided recommendations for improving well-being in the workplace based on findings from Phase 1.

#### **2.5.2** **Research instruments** the research instrument used in Phase 2 consists of 2 tools, as follows

1) **Well-being at work screening checklist.** The theory used to develop the checklist, the characteristics of the checklist, and the interpretation method are described in the research instrument explanation of phase 1.
2) **Focus group questions.** The main question that was used to explore the opinions among participants was, “After you have studied the antecedents for the emergence of work well-being among the LGBTQ + community, what do you think are the appropriate strategies for organizations and government agencies to promote work well-being for this group?” This question aimed to prompt detailed reflections on improving workplace well-being, considering social, emotional, and professional aspects. Participants' responses provided valuable insights into practical policies, programs, and practices to create inclusive and supportive work environments for LGBTQ + individuals, forming the basis for recommended strategies to enhance workplace well-being.

#### 2.5.3 Research procedure

Focus group discussions were conducted around 60–90 min by the principal investigator using Google Meet to analyze the data. The data from the first phase was presented to the first group of six participants to generate strategies to improve workplace well-being in the LGBTQ + context.

#### 2.5.4 Data analysis

The recorded data from the focus group discussion were transcribed verbatim by the research team manually. The data analysis combined deductive and inductive approaches. A deductive approach was initially used to generate main themes based on pre-existing theoretical frameworks. Subsequently, an inductive approach was employed to generate new codes and categories that supported each identified theme, allowing for new insights. This involved open coding to identify significant phrases from the transcribed data to address the research objectives, categorizing these codes into complementary or new sub-themes, and refining the initial themes to reflect the actual data. A constant comparison method was used to ensure consistency and thoroughness in capturing theoretical perspectives and novel insights from the data [24].

The data analysis employed the trustworthiness verification method using method triangulation, which involves examining the consistency between response content and observations. Method triangulation enhances the credibility and validity of qualitative research by cross-verifying data from multiple sources [26]. Additionally, peer debriefing was utilized, wherein two researchers independently analyzed the data. Peer debriefing acts as a form of investigator triangulation, providing an external check on the research process and findings, thereby enhancing the reliability of the analysis [27]. Subsequently, the findings were compared to ensure the accuracy of the data analysis. This method helps to identify any biases and increases the overall trustworthiness of the research [25].

### 2.6 Phase 3

The goal of Phase 3 was to provide feedback on the strategies to improve workplace well-being generated in Phase 2 and evaluate the strategies by evaluation form, which has details of the research methodology as follows.

#### 2.6.1 Participants

Seven participants were purposely selected and included LGBTQ + employees with above-average workplace well-being scores (N = 2), a scholar focused on human resources development (N = 1), scholars specializing in positive psychology (N = 2), and a human rights and social welfare activist with expertise in gender and social welfare issues (N = 2). The participants in the second round of the focus group discussion, all seven individuals, were newly selected and did not participate in the first round of focus group discussions.

#### **2.6.2** **Research instruments** the research instrument used in Phase 3 consists of 3 tools, as follows

1) **Well-being at work screening checklist**. The theory used to develop the checklist, the characteristics of the checklist, and the interpretation method are described in the research instrument explanation of phase 1.
2) **Focus group questions.** The main question was used to explore the feedback on improving the strategies, such as “After you have read the strategies for enhancing workplace well-being for organizations and government agencies, what are your thoughts? Is there any content that should be revised or added, and why?”
3) **Evaluation form** This evaluation form consists of strategies refined based on qualitative data from the second focus group discussion, comprising 11 items. The evaluators are members of the second focus group discussion themselves. The evaluation is a Likert scale with three choices to assess the accuracy and appropriateness of each strategy. The first choice indicates “Incorrect or Inappropriate” with a score of −1, the second choice is “Not Sure” with a score of 0, and the third choice is “Correct and Appropriate” with a score of +1. Subsequently, the scores for each item are averaged. A strategy with an average score of 0.5 or higher is accurate and appropriate, suggesting its potential usability.

#### 2.6.3 Research procedure

The second focus group discussions were conducted around 60–90 min by the principal investigator using Google Meet. The seven participants were presented with the initial strategies from the first focus group discussion to provide improvement recommendations. In addition, the participants from the second focus group provided a consensus rating regarding the strategies' accuracy and appropriateness by the congruence index. For the revised strategies to be deemed acceptable, the revised strategy needed to attain an item objective congruence index of at least 0.5 before a comprehensive strategy version could be finalized and issued.

#### 2.6.4 Data analysis

The data analysis and trustworthiness verification for the second focus group discussions followed a similar approach to the first one. Additionally, data analysis from the evaluation form involved calculating the average scores for each strategy assessed by the 7 participants in the second focus discussion group. Any strategy with an average score exceeding 0.5 will be selected and presented in the research findings as a strategy demonstrating accuracy and appropriateness, suggesting its potential usability.

## 3 Results

### 3.1 General information of participants from Phase 1

The participants' ages ranged from 38 to 45. The average age of the participants is 42 years. They were employees who were hired by government or private agencies in Thailand. Also, they had the mean overall well-being scores at work at a high level or over. The participants’ general information is presented in Table 1.

**Table 1:** General information of participants from Phase 1.

No	Participant	Age	Job title	Gender/Sexuality	well-being at work score
1	ID1	38	CEO of private organization	Transgender woman	31
2	ID2	44	Administrative staff, University dormitory	Tomboy∗	30
3	ID3	37	University teacher	Bisexual	24
4	ID4	39	Private company employee	Tomboy∗	29
5	ID5	45	University lecturer	Lesbian	26
6	ID6	53	Director, Private agency	Cisgender/Gay	25
7	ID7	43	Supporting university staff	Sexuality fluid	25
8	ID8	38	Costume designer	Gay	31
9	ID9	38	Training manager	Pansexual	28
10	ID10	44	Healthcare worker	Transgender woman	28

∗∗ Tomboy is a gender identity found in the Thai context, referring to a woman or girl who expresses masculine traits.

### 3.2 Work well-being defined by LGBTQ+

This part shows the qualitative findings from Phase 1, suggesting that work well-being can be defined as the experience of positive emotions encompassing various dimensions. These dimensions include passion and enthusiasm for work, high levels of work motivation, a sense of self-esteem derived from one's work, the recognition and realization of personal values and the meaning of work, a feeling of connectedness with colleagues through positive relationships, and the presence of support from organizations, external agencies, and government in promoting gender and sexuality equalities. Collectively, these positive emotions contribute to an individual's overall work well-being. The quotations below are examples of comments supporting the above definition and dimensions of work well-being.

*“Ajarn [teacher, Thai term used to address people of respect], it was more about having love for what we do. I believe that the first definition of well-being is ‘love our work.'"* ID1

“Other than career success, work well-being is acceptance from people, from coworkers, and from their circles as well. Something like that." ID3

“I feel that helping others is well-being, simple like that. Like … I get to help them in some ways to learn to protect themselves and provide them protection. Or, if they get infected, we'll refer them to the system to receive treatment. The concept is about helping others; that's my well-being. And, one more thing, I get to work with several organizational networks and several local alliances. This is also well-being, to help each other." ID6

“They are eager to work, er, want to do their jobs, have a smile on their faces when going to the faculty or their workplaces, sort of. And that's what is happening to me. So, work well-being is the same as others; I want to have good relationships with coworkers. I don't want coworkers to have negative perceptions about me." ID8

### 3.3 Antecedents of well-being at work among LGBTQ+

Antecedents of well-being at work among LGBTQ + consisted of three factors.

#### 3.3.1 Intrapersonal factors

Intrapersonal factors refer to factors at the individual level that contribute to work well-being. Four themes associated with intrapersonal-level factors were identified. The first includes workplace practices and behaviors. Participants described the importance of feeling connected to one's work and perceiving it as meaningful, which fosters enthusiasm and determination to achieve work goals. Participants reported being open to learning new things and collaborating with people from diverse backgrounds. Relatedly, they emphasized the importance of demonstrating respect for all employees to create a positive image in the workplace.

“We still feel a passion for this profession, and we have goals that we want to continuously strive towards. It's like waking up every day with the desire to forget, wanting to wake up and forget every day to work, much like the concept of ikigai. Yes, we feel delighted with this aspect of our lives," ID3

“However, do not exhibit behaviors that are inappropriate or unsuitable for the occasion. Perhaps it's because when we work, we tend to adopt a professional persona that may not be as expressive or flamboyant as our usual selves.” ID10

The second intrapersonal factor identified was the importance of self-affirming attitudes and behaviors. Here, study participants described the ability to freely express one's gender identity or sexuality in the workplace and assertively and effectively protect one's rights as contributing to well-being.

“If today, we know who we are and learn to love ourselves and our values, I believe that those emotions surrounding us are just something we pass by … I understand that I am transgender, and I embrace and love myself for being transgender. Therefore, those who are gay must also accept and embrace their sexual identities." ID1

The third intrapersonal factor associated with well-being in the workplace was the ability to manage and cope with experiences of discrimination, including putting effort to prove one's capabilities in the face of insults or rejection, exhibiting resilience in the face of conflict and gender prejudice in the workplace, and engaging in assertive communication when addressing injustice or bullying.

“As an LGBTQ + individual, I acknowledge that there are weaknesses and disadvantages associated with it. However, I still have qualities and achievements that compensate for the negative stereotypes and misconceptions about LGBTQ + individuals. By obtaining academic titles and achieving higher positions in the future, I believe I can demonstrate my abilities and gain acceptance from students." ID5

“In the beginning, I must admit that I wasn't entirely calm when faced with offensive looks or language. Whether it was unintentional slips of the tongue, gossip, or talking behind my back, it bothered me initially. But I have come to realize that these are just passing words and incidents. If we take everything too seriously, life will become difficult and unhappy. It's important to let go and not let these things affect us." – ID9

The fourth intrapersonal factor is having a job that promotes an individual's self-esteem. It refers to individuals with diverse gender identities engaging in professions that involve contributing to the well-being of others or the community, aiming to improve the quality of life. The rewards or returns from pursuing such professions can be used to assist others or show gratitude to those who have been supportive.

“It means that we help them, regardless of the method, to make them aware of self-protection. We provide them with protective equipment. We guide them into the healthcare system for treatment. The concept is that assisting people brings us happiness.” Chen

“'Working and earning money bring us happiness. The happiness of earning money is about working and getting paid. If we work and don't get paid, we won't do it. Money helps us, like providing for our parents.'” ID1

#### 3.3.2 Interpersonal factors

Interpersonal factors are factors at the organizational level or its units that contribute to LGBTQ + well-being in the workplace. These factors can be defined as receiving support, fostering positive relationships with coworkers, authorities, and clients, and engaging in professions that promote well-being for others and society. Interpersonal factors are grouped into five subcategories. The first factor is gaining coworker support. It means receiving support and care and fostering positive relationships with colleagues, supervisors, and service recipients. These factors promote opportunities for advancement in work and the ability to work successfully.

“Having a lot of positive relationships, we sit together and work every day. And then we sit back and relax in the office, sometimes bursting into laughter. It becomes a kind of bond. Even though we might get bored with work sometimes, we still want to come and meet our friends." ID7

The second factor is receiving acceptance of one's gender identity and sexuality from coworkers. It means that LGBTQ + individuals can openly express their identity and sexual preferences to their colleagues and be accepted with respect. There is equality with those who have different gender identities and orientations, and efforts are made to help prevent and address issues of discrimination based on gender identity and sexual orientation within the workplace community.

“It's all about watching out for each other, making sure that no one harms others based on their identity. It means supporting each other. Sometimes, when someone uses a term that is not okay, there may be someone who will speak up and say, 'This term is not acceptable. Don't use it.' We are okay with that. It's like they may not be part of the LGBT community, but they've learned what is appropriate and what is not. It's not about should or shouldn't, but it's for the sake of our professional responsibilities." ID8

The third factor is having LGBTQ + inclusion policies in the workplace, which means that the organization is aware of gender diversity and has inclusive practices that embrace all gender differences. An inclusive environment includes allowing individuals to dress according to their gender identity or expression, considering flexibility in work practices, using gender-neutral titles, providing gender-inclusive restroom facilities, and conducting job recruitment without gender-based discrimination.

“Regarding the dress code at work, they don't require that I wear male uniforms. I always wear women's skirts …. They would say, 'You're a Miss., why do you dress like this?' But in this case, he wasn't strict at all. He's fine with everything. He announces things about me, my name in the workplace, and he always uses 'Khun' [a Thai term used to address people as a form of respect, gender-neutral] when mentioning me." ID10

The Last factor is achieving success in one's work. It means that the performance evaluation results, from self-assessment by individuals with gender diversity and feedback from relevant parties, are effective and align with the desired outcomes. Achieving success at work includes receiving commendation, recognition, awards, or constructive feedback that is beneficial for further development and future success.

“We always keep track of how our work is received, what kind of feedback we get, who talks about our work, and if there's anyone criticizing it. Interestingly, there are very few instances where someone comes directly to criticize. If there's any dissatisfaction with what we do, we usually ask our seniors, actors, close associates, and the team every time. We inquire if it's okay, give us some feedback, how much would you rate it, and well, it's not that bad. It's okay to be criticized; that's part of the process." ID8

**3) Social-ecological factors.** The social-ecological factors refer to the factors at the level of civil society or the government sector that contribute to workplace well-being. These factors can be defined as the support provided by the government sector through policies or laws enacted to promote equality and justice for LGBTQ + individuals, ensuring that they can enjoy equal rights. The social-ecological factors are grouped into two subcategories. The first factor is support from the government sector for promoting equality. It refers to assistance and support from the state, both in terms of policies and legislation, aimed at fostering equality and fairness for LGBTQ + individuals. Governmental sector support encompasses rights related to healthcare, employment, marriage, changing titles, and adopting children to ensure equal opportunities and fairness. The goal is to establish laws and policies that promote equality, impacting both societal and daily life aspects for LGBTQ + individuals. Examples of appropriate policies include creating inclusive and accepting spaces and acknowledging gender differences comprehensively and equally at all levels of governmental or societal organizations within a country.

“They should be generous, like providing medical coverage. They could allocate a quota for sex reassignment surgery or copay. This issue is necessary because it affects well-being. Also, it's psychological … that some individuals may, for instance, want to transition to a different gender. And we must consider it as a gender disorder, right? Thus, treatment should be provided for them … If they want to change their title, let them change. What's wrong with it? And when it comes to getting married, love has no boundaries, right?" ID10

The second factor is support from external agencies for LGBTQ + people. It means support from social or private organizations in promoting a positive image of LGBTQ + individuals to the public. Appropriate support includes advocating for rights during instances of discrimination, being a voice for LGBTQ + individuals in society, and contributing to creating a just and equitable society for those with gender and sexual diversity.

“If it's legally established and not-for-profit, I believe it should exist. For example, when we need help or want to file a complaint, we can seek assistance from this unit. They have knowledge about the law and can help us. I believe that it is useful and should be legalized to provide space for individuals to make complaints and ask for help." ID5

### 3.4 General information of participants from phases 2 and 3

Demographic data of the 13 participants from Phases 2 and 3 are presented in Table 2.

**Table 2:** Demographic data of the 13 participants from Phases 2 and 3.

No.	Phase	Participant	Age	Occupation	Group discussion participation
1	2	ID7	43	Supporting university staff	LGBTQ + employees
2	2	ID10	44	Healthcare worker	LGBTQ + employees
3	2	ID11	43	University lecturer	Positive psychology scholars
4	2	ID12	43	Counseling psychologist	Positive psychology scholars
5	2	ID13	37	Human Resources Personnel at a Private Company	Human resources development scholars
6	2	ID14	38	Director of Foundation for LGBTQ + Support	Human rights and social welfare activists on gender and social welfare issues
7	3	ID1	38	CEO of private organizations	LGBTQ + employees
8	3	ID8	38	Costume designer	LGBTQ + employees
9	3	ID15	35	University lecturer	Positive psychology scholars
10	3	ID16	41	University lecturer	Positive psychology scholars
11	3	ID17	35	University lecturer	Human resources development scholars
12	3	ID18	35	The Foundation for Service Workers' personnel	Human rights and social welfare activists on gender and social welfare issues
13	3	ID19	43	Psychologist and lecturer	Human rights and social welfare activists on gender and social welfare issues

### 3.5 Recommendation to improve work well-being for LGBTQ + adults

The recommendations for enhancing work well-being gathered from both focus group discussions can be categorized into strategies for organizations, civil society, or government.

1) **Strategies for organizations designed to increase the work well-being of LGBTQ + employees** are divided into six categories as follows:
  1.1) The organizations/agencies' core values should emphasize diversity and equity. These core values will enable everyone to treat each other equitably in the workplace.

“This makes change easier by defining values as employees' priority. Let's say that here, equity is a value. And the set time is the most important thing right away. Then, it asserts that the organization's success in this area of equity will depend on how the individuals in it perform. A certain behavior (one, two, three, four, and five) will happen; this is how you show that you are equitable. Or such behavior for first, second, third, fourth, and fifth is how unequal individuals perform. Well, I think this will be a part that will help with organizational policy.” - ID11

1.2) Arranging workshops to promote understanding of equality and equity for all employees. Organizations must consistently provide training on equality and diversity issues, encompassing gender, sexual orientation, disability, and ethnicity, to their employees. Training ensures that employees are sensitive and embrace the diversity of their colleagues.

*“It is necessary, indeed. Many companies, particularly multinational corporations in Thailand, have begun training their employees annually to raise awareness of workplace diversity. They have implemented guidelines and policies that support employees of all nationalities who come to work in Thailand. This makes employees feel valued and truly appreciated by the company, emphasizing a strong commitment to equality.”* ID17

1.3) Organizations/agencies should allow employees to choose work attire that corresponds to their talents so that individuals of diverse gender identities can live fulfilling lives.

“People have different talents and abilities. Katoey should have the opportunity to work in roles that make use of their specific skills, like caregiving for the sick, creating something fashionable, rather than assigning tasks based on their sex assigned at birth, which may not match their abilities." - ID8

1.4) Participants addressed the facilitation of LGBTQ + individuals. Transgender employees should be permitted to use restrooms according to their gender identity. Shared restrooms should be created to attain equality, reduce bias, and remove restrictions on LGBTQ + individuals from utilizing restrooms.

“Some katoeys [the term "katoey" refers to individuals who are transgender or have a non-binary gender identity, often specifically referring to transgender women.] have a tough time using the restroom at work. If they use the men's room, it can make guys uncomfortable. If they use the women's room, some women might not like it. That's why it's a good idea to have restrooms that anyone can use comfortably. This shows that the company cares about everyone, no matter their gender." - ID1

1.5) The organizations/agencies should provide LGBTQ + personnel with opportunities to obtain training and conduct work that supports or enriches others. This strategy will encourage employees to increase their self-esteem and well-being at work.

“I'm a transgender person and work as staff at the foundation. No one believed that an uneducated transgender person would be able to provide guidance and support in the community. I turned into a trans, perhaps considered a service worker. However, I have been properly trained by senior members of the organization, mentors who have become mentors as donors or ministry officers. I take care of LGBTQ + service workers as a coach or mentor for them. I'm the equivalent of a doctor or nurse in my treatment style.” - ID14

1.6) The organizations/agencies should provide leave benefits for transgender employees to have gender-affirmative surgery to achieve equality with cisgender who request maternity leave and ordination leave.

“I saw the H.R. policy, and there's nothing related to transgender benefits. We know why women are women and men are men, but why are some individuals transgender? I called for an H.R. meeting and discussed, for instance, if I decide to undergo gender-affirmative surgery for 30 days. Because of the major surgery, no transgender person after the operation could work because it's probably so hard for any activity, even sitting down. If one has breast surgery, one must take a week off, and H.R. must pay them for that week. However, according to the Ministry of Labor, there is no such policy, but it would be beneficial to have the applicants informed upon the interview recruitment.” - ID14

1.7) Organizations/agencies should also offer support to the families of LGBTQ + employees. These families include the employee's partner, who cannot legally register their marriage, or an adopted child. This support is essential to maintain parity with employees of the predominant gender or sexual orientation.

“In a university that provided the COVID-19 vaccine, couple protection is offered. It also covers partners of LGBTQ + persons; for instance, if you were gay and resided in the same house as your loved one. Even though the law has not yet been issued, having a life partner in the same residence is not supported by any laws. The university staff then created a certificate and sent it to the dean. That is the name of this lifetime companion. This information was entered into the university's vaccine allocation system in this manner, in addition to establishing policies or offering services to LGBTQ + individuals individually. We do not know how quickly the government will introduce legislation in the future. Nevertheless, is it possible for the organization to emphasize this while offering all the others? Consider having a partner for individuals with gender diversity as well.” – ID7

2) **Strategies for civil society or government in enhancing well-being at work for LGBTQ + employees** are divided into three topics as follows:
  2.1) Most participants stated that the government should establish a direction for the nation's governance that supports LGBTQ + equality by enacting laws addressing equity and LGBTQ + equal rights. They advocated for tangible achievements that will make people of gender diversity content in their lives.

“Assuming our LGBTQ + life partner becomes very ill and needs medical care, can we sign for them? Due to their obligations, they must visit the doctor. May we sign the documents? Or require someone to sign? Since we have no registration, we lack the legal authority to care for our partner. Suppose government policy at the national level can persuade people that it is acceptable for everyone to have equal rights without gender discrimination. In that case, we will all have true equity. And the law can be extensive, covering diversity with respect. I feel we will generally be happier." -ID12

2.2) The governments or civil society should provide hormone services, cosmetic surgery, or gender affirmative surgery to transgender people as part of government welfare.

“We want to overcome these barriers by using hormones or surgery as the key to happiness, or in other words, well-being." (ID14)

2.3) Participants suggested that the government establish an official agency to generate knowledge syntheses and educate the public on gender/sexual diversity concerns. This agency would promote the holistic health of LGBTQ + individuals and give them a voice to protect LGBTQ + employees from discrimination in the workplace. It would also work to address mental health issues and concerns associated with an individual's sexual orientation or gender identity promptly and with simplicity in the application process for treatment to minimize the impact of damage and prevent future harm.

“There must be some form of organization. Establish themselves as one of the voices. Accept accusations, then push them to the limit. It would be excellent to reform the policy at the municipal level or in Thailand. That is key; if it shifts, it will straighten up the circumstances, and the document for local enforcement will be applied. I believed it would be beneficial and actualize. There must be an organization and an agency to file and receive complaints and push these projects forward. Which I believe is significant and must have a method or a mechanism that can obtain, not be slow, know where to remain, and be able to follow through.” - ID14

2.4) The government should endorse the utilization of multimedia across various channels to enhance LGBTQ + awareness among the general public.

*“This could involve disseminating knowledge related to gender and sexual orientation, addressing gender-based violence, supporting organizations that assist victims of workplace and daily life gender discrimination, as well as using media to promote a positive attitude among the public towards the LGBTQ + community and avoiding stereotypes”* – *ID18*

### 3.6 Evaluation of the strategies

All 11 strategies were sent back and assessed by the second focus group, comprising seven participants, who evaluated the Index of Congruence (IOC). The results showed that 11 strategies identified had an IOC value ranging from 0.8 to 1. Scores in this range suggest a high level of congruence. This score suggests high content validity between the strategies identified and the goal of identifying strategies for improving workplace well-being.

## 4 Discussion

This research examined the concept of workplace well-being for members of the LGBTQ + community. Specifically, the study sought to identify the factors contributing to workplace well-being. The results showed that positive emotions, such as motivation and support for gender and sexuality equality from organizations, external agencies, and the government, influence workplace well-being. In addition, the study revealed that intrapersonal, interpersonal, and social-ecological factors influence workplace well-being among LGBTQ + employees.

The research findings on the meaning of workplace well-being align with Bartel's workplace well-being model, which suggests that well-being at work is best achieved when employees feel a two-dimensional sense of meaning and purpose and experience positive social interactions [15]. However, the meaning of workplace well-being in this study has expanded beyond Bartel's definition to encompass the view of LGBTQ + participants, who see workplace well-being as including support from organizations, external agencies, and the government in promoting gender and sexuality equality. This expanded definition is supported by findings from the International Labour Organization [6], which highlight the importance of institutional support in mitigating workplace discrimination and enhancing well-being among LGBTQ+.

Additionally, when considering the antecedents of workplace well-being, which consist of intrapersonal, interpersonal, and social-ecological factors, it aligns with other studies discussing well-being factors in different populations where well-being should cover internal and external dimensions of individuals [23,28]. For example, research by Livingston, Jackson-Nevels, & Reddy [29] highlights the role of social, cultural, and economic status in contributing to well-being. Similarly, the United Nations Development Programme [7] emphasizes the need for comprehensive support systems, including governmental policies, to foster workplace well-being for LGBTQ + individuals.

When examining the meaning of all three main factors of workplace well-being in this study, specific aspects within the LGBTQ + group were identified. These include the importance of self-affirming attitudes and behaviors, the ability to manage and cope with experiences of discrimination, receiving acceptance of one's gender identity and sexuality from coworkers, having LGBTQ + inclusion policies in the workplace, and support provided by the government sector through policies or laws enacted to promote equality and justice for LGBTQ + individuals, ensuring that they can enjoy equal rights. The definition of these three factors can be considered as new knowledge generated from this study.

The research findings on workplace well-being also align with the social-ecological framework, emphasizing the importance of interpersonal and intrapersonal factors in shaping well-being [18]. Similar studies conducted in Thailand also suggest that the development of well-being among the LGBTQ + population should consider factors covering intrapersonal, interpersonal, and social-ecological dimensions [9,12]. For instance, Meyer's minority stress model [30] underscores how social structures and interpersonal relationships affect the mental health and well-being of marginalized groups, including LGBTQ + individuals.

The study highlights the necessity of implementing supportive strategies to benefit LGBTQ + individuals, including backing from organizations and external and government sectors. These strategies span organizational and governmental realms. Some critical strategies identified involve embedding diversity and equity in organizational values, conducting equality workshops, offering leave benefits for transgender employees seeking gender-affirmative surgery, enacting laws supporting LGBTQ + equality, and providing essential services like hormone therapy and gender-affirming surgery as part of governmental welfare. These initiatives collectively aim to create a more inclusive and supportive environment for LGBTQ + individuals within workplaces and broader society. This finding aligns with the model of Bond & Haynes [18], highlighting the necessity to address or develop individuals with the social-ecological framework. Additionally, this research finding aligns with the UNDP study [9] and Perales [31], which recommends that organizations and governments should enact necessary regulations, legal, administrative, training, and other measures to prevent discrimination against LGBTQ + individuals and to advance their rights and liberties. Other studies have reported that most LGBTQ + individuals do not have access to fundamental rights and equal employment opportunities and treatment [[6], [7], [8],^10^]. Therefore, it is crucial to protect LGBTQ + rights through legislation to encourage their full development.

## 5 Limitations and future research

The limitations identified in this study have important implications for future research on LGBTQ + workplace well-being. The potential selection bias inherent in the qualitative nature of this research suggests that the experiences of some gender identities and sexual orientations, such as Queer, Transman, Non-binary, and Asexual individuals, may not be adequately represented. This underrepresentation limits the ability to draw comprehensive conclusions about the diverse experiences within the LGBTQ + community. Therefore, future studies should include a broader and more varied range of participants to capture better the full spectrum of experiences and identities within the LGBTQ + population.

Moreover, the focus on participants in middle adulthood means that the unique challenges and perspectives of LGBTQ + individuals in the early stages of adulthood, particularly those who are just entering the workforce, are not fully explored. This gap highlights the need for research that includes younger LGBTQ + individuals to understand how their early career experiences shape their workplace well-being and professional development.

These limitations suggest that while the findings of this study provide valuable insights into the workplace well-being of LGBTQ + individuals, they may not be fully generalizable to the entire LGBTQ + population. Future research should strive to address these gaps by employing more inclusive strategies and expanding the demographic range of participants. By doing so, researchers can develop a more comprehensive understanding of the diverse experiences and challenges faced by LGBTQ + individuals in various stages of their careers and across different gender identities and sexual orientations.

Implications for future research also involve emphasizing the significance of community engagement activities aimed at gaining deeper insights into the needs of LGBTQ + individuals and developing effective strategies to enhance workplace well-being. To ensure cultural appropriateness and alignment with the recommendations provided, it is imperative to include LGBTQ + members as consultants and research team members in these projects. Their involvement will facilitate a more thorough understanding of the community's perspectives and contribute to developing tailored interventions.

## 6 Conclusion

This qualitative inquiry aimed to provide an in-depth understanding of the well-being of a highly underserved segment of the workforce in Thailand. Utilizing community-engaged approaches, we identified factors influencing workplace well-being across various intrapersonal, interpersonal, and social-ecological levels. Further, comprehensive strategies for promoting safety, inclusion, and, ultimately, well-being were identified. Further efforts are needed to implement the identified approaches as potential strategies for creating a more inclusive workplace for LGBTQ + employees.

## CRediT authorship contribution statement

**Nanchatsan Sakunpong:** Writing – review & editing, Writing – original draft, Visualization, Validation, Supervision, Resources, Project administration, Methodology, Investigation, Funding acquisition, Formal analysis, Data curation, Conceptualization. **Ramida Mahantamak:** Writing – review & editing, Investigation, Formal analysis. **Pilaiporn Sukcharoen:** Writing – review & editing, Investigation. **Alicia K. Matthews:** Writing – review & editing, Supervision.

## Consent

Written informed consent was obtained from all participants. Before signing, the participant read the research details, including the research objectives and the name and background of the research team, and then decide to sign. The names of the participants were not identified to ensure the confidentiality of this research, as stated in the informed consent. The participants consented to the publishing of all data included in the manuscript.

## Ethical approval

The Institutional Review Board (IRB) of Srinakharinwirot University reviewed and approved this study on May 25, 2020 (Protocol No.SWUEC/E−162/2563).

## Data availability statement

The authors do not have permission to share data.

## Funding disclosure

This research was supported by funding from the 10.13039/501100020818Strategic Wisdom and Research Institute of 10.13039/501100007280Srinakharinwirot University. However, the funders played no part in the study design, data collection and analysis, publication decision, or manuscript preparation.

## Declaration of competing interest

The authors declare that they have no known competing financial interests or personal relationships that could have appeared to influence the work reported in this paper.
